# Interventional Pain Management in Multidisciplinary Chronic Pain Clinics: A Prospective Multicenter Cohort Study with One-Year Follow-Up

**DOI:** 10.1155/2017/8402413

**Published:** 2017-10-15

**Authors:** Cláudia Gouvinhas, Dalila Veiga, Liliane Mendonça, Rute Sampaio, Luís Filipe Azevedo, José Manuel Castro-Lopes

**Affiliations:** ^1^Department of Biomedicine, Faculty of Medicine, University of Porto, Porto, Portugal; ^2^Chronic Pain Center-Anesthesiology Department, Centro Hospitalar do Porto, Porto, Portugal; ^3^National Observatory for Pain (NOPain), Porto, Portugal; ^4^Institute for Molecular and Cell Biology (IBMC), University of Porto, Porto, Portugal; ^5^Department of Community Medicine, Information and Health Decision Sciences (MEDCIDS), Faculty of Medicine, University of Porto, Porto, Portugal; ^6^Centre for Health Technology and Services Research (CINTESIS), University of Porto, Porto, Portugal

## Abstract

**Background:**

Interventional Pain Management (IPM) is performed in multidisciplinary chronic pain clinics (MCPC), including a range of invasive techniques to diagnose and treat chronic pain (CP) conditions. Current patterns of use of those techniques in MCPC have not yet been reported.

**Objective:**

We aimed to describe quantitatively and qualitatively the use of IPM and other therapeutic procedures performed on-site at four Portuguese MCPC.

**Methods:**

A prospective cohort study with one-year follow-up was performed in adult patients. A structured case report form was systematically completed at baseline and six and 12 months.

**Results:**

Among 808 patients referred to the MCPC, 17.2% had been prescribed IPM. Patients with IPM were on average younger and had longer CP duration and lower levels of maximum pain and pain interference/disability. The three main diagnoses were low back pain (*n* = 28), postoperative CP, and knee pain (*n* = 16 each). From 195 IPM prescribed, nerve blocks (*n* = 108), radiofrequency (*n* = 31), and viscosupplementation (*n* = 22) were the most prevalent. Some IPM techniques were only available in few MCPC. One MCPC did not provide IPM.

**Conclusions:**

IPM are seldom prescribed in Portuguese MCPC. Further studies on IPM safety and effectiveness are necessary for clear understanding the role of these techniques in CP management.

## 1. Introduction

Chronic pain (CP) is a worldwide public health problem that causes a substantial burden on healthcare systems and society, taking into account its high prevalence, economic costs, and the quality of life impairment of the patients and their families. CP requires a multidisciplinary approach for its adequate assessment and management [1–5]. For more than twenty years, the International Association for the Study of Pain (IASP) defined chronic CP as “pain that persists beyond normal tissue healing time, which is assumed to be 3 months” [6]. Variations in the definition of CP between and within countries, differences in standards of living and healthcare resources, high prevalence of pain-generating diseases, cultural background, and local traditions can help explain the estimated CP prevalence ranging from 11% to 55% [2, 7–9]. In a recent epidemiological study, CP prevalence in Portugal was estimated at 37% [10]. CP of moderate to severe intensity has been estimated to occur in 19% of European adults and 40% of them reported inadequate management of their pain [8, 11].

CP is one of the most common reasons why people seek medical care [1]. The economic impact of CP in the USA, in 2008, ranged from $560 to $635 billion USD [12]; in Portugal, it was €4611.69 million (in 2010), corresponding to 2.71% of the Portuguese annual gross domestic product, with 42.7% direct and 57.3% indirect costs [2]. Indirect costs of CP are mainly linked to work absenteeism with reduced levels of productivity and increased risk of leaving the labor market [13].

Ideally, supported by the most recent guidelines for the CP management, these patients should have access to a multidisciplinary range of diagnostic and therapeutic modalities appropriate to their clinical condition [14–17]. The primary emphasis should be placed on pain management to improve physical and social function and minimize the disability [18–20]. Pain management includes pharmacological therapy, psychological assessment and treatment, invasive techniques, occupational therapy, and rehabilitation medicine [2, 15, 20–25]. Interventional Pain Management (IPM) encompasses a wide variety of techniques such as epidural injections [26–29], nerve blocks [18, 21, 30], joint infiltrations [31, 32], spinal cord stimulation [33, 34], botulinum toxin application [35–37], and radiofrequency denervation [38, 39]. Recent data suggests that some of the IPM techniques allow a superior improvement in physical and functional status of CP patients [18, 25, 26, 35, 38], as well as a reduction in associated work absenteeism [18]. Other therapeutic procedures (OTP) are also performed on-site at multidisciplinary chronic pain clinics (MCPC), namely, acupuncture [40–46] and infusion of parenteral drugs [47–49].

In Portugal, there is no available information regarding patterns of prescription of IPM or OTP performed on-site at MCPC. Therefore, as a part of a larger real-world outcomes research prospective cohort study on CP patients followed at MCPC, this study inquired about the utilization of those techniques and procedures. We aimed at describing quantitatively and qualitatively the patterns of prescription of IPM and OTP performed on-site at MCPC in Portugal.

## 2. Methods

### 2.1. Selection and Description of Participants

Participants were recruited at their first consultation in four MCPC from the North of Portugal, in the context of a large real-world outcomes research project. The eligible population included adult patients (≥18 years old) with chronic noncancer pain lasting more than 3 months (the standard IASP criteria for CP [50]) or with cancer pain regardless of its duration. Patients with psychiatric and/or cognitive impairment, unable to communicate verbally or not fluent in Portuguese were excluded. Participants were followed for twelve months and their clinical records were obtained at baseline and six and 12 months. From all 1343 patients eligible, 808 patients were elected to be part of this study. From these, 139 patients had IPM at least once during the one-year follow-up (Supplement 1 in Supplementary Material available online at https://doi.org/10.1155/2017/8402413).

All participating patients were previously informed of the study objectives and about the data selection and collection procedures, and questions regarding the study, posed by the patients, were properly answered by the research team. All participating patients signed an informed consent form. The study protocol was approved by institutional review boards, ethics committees of the participating hospitals, and CNPD, the Portuguese Data Protection Authority.

### 2.2. Instruments and Variables

A structured questionnaire was used in baseline interviews. For the purpose of the present study the following data were systematically collected: demographic characteristics, clinical and pain characteristics, and IPM and OTP performed on-site at MCPC prescribed. The Brief Pain Inventory (BPI) [51] which is recommended by consensus groups in the area of measurement and evaluation of pain [52] was used. This instrument was constructed for measuring and evaluating pain in a multidimensional perspective. It includes 15 items evaluating pain presence, severity, location, functional interference, therapeutic strategies, and efficacy of pain management. It is easily applicable, fast, and simple and with very good psychometric properties [53–56]. For the present study, only two constructs were assessed: pain severity and functional interference. Pain severity scale entails four items, maximum, minimum, on average, and at this moment, rated with a numerical scale (0 [no pain] to 10 [the worst pain]) and categorized using Serlin et al. classification [57]. The functional interference scale consists of seven items with numerical rating scale (0 [no interference] to 10 [extreme interference]) that assess pain interference in general activities, mood, mobility, work, personal relationships, sleep, and pleasure to live.

In the descriptive analysis of the sample (all patients) and subsamples (patients with and without IPM), we defined the general characteristics, including sociodemographic (sex, age, educational level, and family support), professional/occupational status, pain characteristics (duration, persistence pattern, location, severity, and interference), general health, and clinical variables (International Statistical Classification of Diseases and Related Health Problems- (ICD-) 10 diagnostic classification). The persistence pattern of pain was classified as continuous, pain present every day/always, or discontinuous (recurrent [pain present once to several times/week] and sporadic [pain present less than once to several times/month]).

### 2.3. Statistical Analysis

Categorical variables were described as absolute (*n*) and relative (%) frequencies; continuous variables were described using the median and interquartile ranges considering the tested asymmetric (not normal) distribution or mean and standard deviation otherwise. We used the Chi-square test, or the Fisher's exact test, to test hypotheses for categorical variables. In the case of continuous variables (with asymmetrical distribution) the Mann–Whitney test was used. For all hypothesis tests a significance level of *α* = 0.05 was considered. The statistical analysis was performed using the software SPSS v21.0®.

## 3. Results

Table 1 shows the number of patients that were prescribed IPM and/or OTP throughout the study. Considering IPM, nerve blocks were the most frequently prescribed at all time points (39 patients at baseline, 32 patients at 6 months, 20 patients at 12 months, and 88 patients in total throughout the one-year follow-up). On the contrary, botulinum toxin and viscosupplementation were prescribed to a few patients and almost exclusively at baseline. Neuromodulation by neurostimulation was only prescribed to one patient at 6 months. OTP were also prescribed to a considerable number of patients. Among them, acupuncture was prescribed to 52 patients throughout the study, followed by drug infusions to 31 patients.

Sociodemographic characteristics of all patients, patients with IPM, and patients without IPM are described in Table 2. It was observed that 17.2% of the patients followed in MCPC had IPM prescribed, of which 71.9% were women and 28.1% men. Moreover, age was significantly lower in patients with IPM (54.6 (44.0–65.0) versus 59.7 (50.0–71.0) years, *p* < 0.001). Elderly patients (over 75 years) were less frequently prescribed IPM. Patients with IPM were mainly full- or part-time workers while those without IPM were mostly retirees (*p* < 0.001).

Table 3 shows pain clinical characteristics, at the inclusion time in our study, among all patients, patients with IPM, and patients without IPM. Almost 1/3 of the patients with IPM had pain for over 10 years, while almost 90% of patients with pain duration from 3 to 12 months did not have IPM (*p* = 0.006). Patients in both groups referred to almost exclusively continuous pain. Significant differences in pain intensity could only be seen for pain at its worst (8.01 without IPM versus 7.23 with IPM; *p* = 0.003). The location of the pain was heterogeneous and statistically significant differences were found; in all patients that indicated pain in unspecified bones, joints, and muscles 92.1% were patients without IPM, whereas among all cases of pain in inguinal and pelvic region 31.2% are reported by patients with IPM (*p* = 0.047). Patients with IPM had significantly lower pain interference score (*p* < 0001). Specifically, pain-related interference was inferior in general ability (6.20 ± 3.26 versus 7.04 ± 2.71, *p* = 0.015), walking ability (5.76 ± 3.81 versus 6.67 ± 3.44, *p* = 0.024), normal work (6.09 ± 3.53 versus 7.26 ± 2.82, *p* < 0.001), relations with other people (3.17 ± 3.64 versus 4.22 ± 3.58, *p* = 0.002), and enjoyment of life (4.18 ± 3.95 versus 5.55 ± 3.79, *p* < 0.001). More than 57% of patients who had no pain interference had invasive techniques for pain management, while 88.1% of patients with severe interference did not had IPM prescribed. In general health no differences were found.

Using the ICD 10 to classify patient's pathologies, 34 diagnoses were obtained from all patients. The five most prevalent diagnoses in patients with IPM were low back pain (28 patients), chronic postoperative pain (16 patients), knee pain (16 patients), pelvic and perineal pain (13 patients), and low back with sciatica (11 patients). We only found five significant differences between patients with and without IPM in what concerns the diagnosis. IPM was only used in 2.8% of patients with fibromyalgia (*p* = 0.013) and 4.2% of patients with cancer pain (*p* < 0.001), while it was used in 36.7% of patients with low back pain with sciatica (*p* = 0.011), 41% of patients with knee (*p* < 0.001) pain, and 46.4% of patients with pelvic or perineal pain (*p* < 0.001) (Supplement 2).

Throughout the study 420 treatments were prescribed as seen in Table 4. The most prescribed IPM in all assessment time points was nerve blocks. In one-year follow-up nerve blocks were 108 times prescribed followed by radiofrequency (31 prescriptions) and viscosupplementation (22 prescriptions). The botulinum toxin and viscosupplementation prescriptions were scarce and occurred almost exclusively at baseline. The most prescribed OTP performed on-site at MCPC at baseline were infusions (43 prescriptions) and at 6 and 12 months acupuncture (73 and 45 prescriptions, resp.).

The five most frequent diagnoses of patients that were prescribed each of the IPM and/or OTP are shown in Table 5. IPM was not exclusive to patients with a specific diagnosis. Nerve blocks, epidural, and radiofrequency were extensively used in several diagnoses, and the same occurred with acupuncture. Botulin toxin was prescribed to 69.2% of patients with pelvic and perineal pain; viscosupplementation was prescribed to 68.4% of patients diagnosed with knee pain. Considering OTP performed on-site at MCPC, drug infusions were prescribed to 57.9% of patients with cancer pain.

There was large heterogeneity in the type of treatments and prescription frequency in each MCPC. It is noted that there are techniques only prescribed in some MCPC, such as viscosupplementation, radiofrequency, botulinum toxin, and neurostimulation. One MCPC had only OTP performed on-site (Supplement 3).

## 4. Discussion

There are very few studies focused on IPM utilization patterns in multidisciplinary CP clinics [17, 24]. To our best knowledge, this is the first study focused on this issue in Portugal and one of the very few elsewhere.

We found that nerve blocks were the most frequent IPM prescribed in MCPC; with a total of 420 IPM or OTP prescribed during the one-year follow-up. A total of 139 patients (17.2%) in our cohort received IPM for their CP management. Of those, almost 32.4% had pain for over 10 years and 92.1% of these reported a continuous pain pattern. There was a diversity of IPM prescribed in the participating MCPC, and botulinum toxin, viscosupplementation, neurostimulation, and radiofrequency were only available in very few centers. In our cohort there is a higher prevalence of women with CP which is in accordance with the available literature [10, 20, 58], and average pain intensity was approximately 6 in a 0–10 numerical rating scale.

In general, the success of these techniques is partly dependent on the experience and skill of the provider. There is controversy over the evidence that supports these techniques [16], as there is no sufficient data to ascertain the efficacy of some procedures. In some cases, pain relief is only observed during a short period and does not essentially convert into improved function and reduced work absenteeism [59]. Moreover, there is a risk of potential complications associated, but most are transient and often subclinical [30]. IPM techniques have been used for a long time as a last option for patients when all other conservative approaches have failed. Therefore, it is understandable that patients to whom they are actuality prescribed have a much longer reported pain duration [59].

In the present study, IPM were prescribed mainly to patients that did not report significant interference of pain, as measured by the functional interference scale of the Brief Pain Inventory, while the vast majority of patients that reported severe interference were not prescribed IPM. The reasons for these unexpected results are not clear at present, and one can only speculate that patients with severe interference suffered from conditions and/or had a health status (e.g., comorbidities) less likely to be treated by IPM, but additional data is required to investigate this issue.

Among the great variety of IPM used in multidisciplinary pain management [14, 25], six techniques plus three OTP performed on-site at MCPC were prescribed in the course of our study.

Nerve blocks, the most frequently prescribed technique in the present study (88 patients), consist in anesthetics delivered to visceral and peripheral nerves in order to interrupt nociceptive input at the source of pain [16, 28]. This procedure may be understood, especially by patients, as curative, but similarly to other IPM techniques, that is not the case. However, it can be very useful, for instance, to alleviate pain and allow patient to take part in their physical therapy [30].

Viscosupplementation consists of intra-articular injection of hyaluronic acid derivatives. It is one of the more used local treatments for osteoarthritis along with corticosteroid injection. It has been used mainly in knee joint, where it shows moderate and significant pain reduction and increased function [32], but a recent meta-analysis provided no evidence that the effect remains longer than six months [31].

Radiofrequency denervation consists in nerve ablation using heat generated by a radiofrequency current [38]. Facet joints radiofrequency is one of the most prevalent indications for its use with high rates of clinical improvement. However, these effects have also a limited temporal efficacy [25, 39].

Botulinum toxin injections as well as acupuncture and dry needling techniques are effective options for myofascial pain, namely, trigger point's deactivation [36, 38]. However there is no clear evidence about long-term effectiveness of botulinum toxin treatment [35].

Epidural injection was primarily recommended for radicular pain from a herniated disc but could also be performed in patients with spinal stenosis and other low back pain conditions [25, 26]. Fair evidence of moderate benefit versus placebo injection for short-term low back pain relief was found [27], and even when compared with other pharmacological treatment, epidural injection may be better in some outcomes, but the differences are transient and modest [28]. In fact, epidural steroid injections are the most commonly performed procedure in USA, differing from what we have found in our study, yet evidence is controversial and inconclusive as to their long-term effectiveness [25, 28, 29]. The potential complications associated with neuraxial techniques performance may explain the low prevalence reported on its use.

Neuromodulation is used in some Portuguese MCPC. However, during the recruitment period of our study there was only one patient who was referred to this treatment [33]. Spinal cord stimulation has formal indication in CP conditions such as failed back surgery, complex regional pain syndrome, peripheral vascular diseases (Buerger disease, Raynaud syndrome, and limb ischemia I/II), and refractory* angina pectoris* [34].

Taking into consideration OTP performed on-site, prescribed in the MCPC, acupuncture was the most frequently prescribed. Some evidence supports acupuncture effectiveness for chronic low back pain treatment [41], fibromyalgia [42], and neck pain treatment [43]. It is increasingly prescribed [40], but the lack of strong studies that clearly prove its effectiveness has delayed its global recognition [44–46].

Pain is a common symptom in cancer patients. In spite of advances in pain management, effective pain control remains an ongoing challenge, despite high doses of opioids [47]. Drugs infusions can be used safely and should be considered to manage cancer neuropathic pain [47, 48]. Trigeminal neuralgia is the most common neuralgia in which intravenous infusion of lidocaine may be a therapeutic option when other treatments are ineffective [49]. Thus further investigation will be needed to evaluate clinical significance of infusion therapy [48].

The present findings clarify which and in what extent IPM and OTP performed on-site at MCPC are being prescribed. This study has several strengths, namely, inclusion of several MCPC, a well-structured and detailed questionnaire, telephonic interviews performed systematically, a restricted and trained team, a large sample, and a 12-month follow-up period. The data presented are unique and to the best of our knowledge there are no similar data in other MCPC that describe their current clinical practice and are useful for comparison and benchmarking.

We have a comprehensive and systematic description of IPM and OTP performed on-site, prescribed, and actually used in MCPC, constituting the clear added value of our study. However, some IPM can be performed for therapeutic or diagnostic purposes and this has not been differentiated in the present study. Further ongoing research will provide data on the evaluation of real-world effectiveness and patient's reported satisfaction by type and timing of techniques performed.

## 5. Conclusions

In Portugal IPM is available in most MCPC, but with marked diversity in the types of techniques and clinical indications. IPM is still seldom prescribed, with only 17.2% of patients having IPM as part of their management. Future studies focused on patient's reported effectiveness and satisfaction are needed.

## Supplementary Material

Supplement 1: Methodological flowchart of patients' selection. Supplement 2: Diagnostic classification of all patients, CP patients with IPM and CP patients without IPM. Supplement 3: Prescribed IPM and OTC performed on-site at multidisciplinary chronic pain clinics by multidisciplinary chronic pain clinics, at baseline, six months and twelve months.

## Figures and Tables

**Table 1:** Number of patients that received a prescription of interventional pain management and other therapeutic procedures performed on-site at multidisciplinary chronic pain clinics.

	Baseline (*n* = 808)		Six months (*n* = 801)		Twelve months (*n* = 744)		Total throughout the one-year follow-up (*n* = 744)	
IPM and/or OTC performed on-site								
Yes	89		122		139		—	
No	719		679		605		—	

	*n*	%	*n*	%	*n*	%	*n*	%

IPM								
Nerve blocks	39	4.8	32	4.0	20	2.7	88	11.8
Viscosupplementation	18	2.2	1	0.1	1	0.1	16	2.2
Radiofrequency	13	1.6	11	1.4	5	0.7	28	3.8
Botulinum toxin	10	1.2	2	0.2	1	0.1	12	1.6
Epidural injections	9	1.1	9	1.1	1	0.1	17	2.3
Neurostimulation	—	—	1	0.1	—	—	1	0.1
Total	89	11.0	56	7.0	28	3.8	162	21.8
OTP performed on-site								
Acupuncture	33	4.1	20	2.5	12	1.6	52	7.0
Infusions	21	2.6	8	1.0	11	1.5	31	4.2
Mesotherapy	1	0.1	1	0.1	—	—	2	0.3
Total	55	6.8	29	3.6	23	3.1	85	11.4

At the top of the table, in each row there is the cumulative number of patients who had interventional pain management and/or other therapeutic procedures prescribed at multidisciplinary chronic pain clinics at each assessment time point. *n*, number of patients at each assessment time point. Furthermore, in each row there are the number of patients (*n*) and proportions of patients (%) that were prescribed interventional pain management or other therapeutic procedures performed on-site at multidisciplinary chronic pain clinics at each assessment time point and during the follow-up time. IPM, interventional pain management. OTP, other therapeutic procedures.

**Table 2:** Sociodemographic characteristics of all patients, chronic pain patients with interventional pain management, and chronic pain patients without interventional pain management.

	All ^†^CP patients		CP patients without IPM		CP patients with IPM			^*∗*^ *p*
	*n*	%	*n*	%	*n*	%	UF (%)	
Gender								
Male	265	32.8	226	33.8	39	28.1	14.7	0.199
Female	543	67.2	443	66.2	100	71.9	18.4	
Total	808	100.0	669	100.0	139	100.0	17.2	

Age								
Median (P25–P75)	60.0 (48.3–70.8)		59.7 (50.0–71.0)		54.6 (44.0–65.0)			**<0.001**

Age categories								
18–24 years	9	1.1	5	0.7	4	2.9	44.4	**0.010**
25–34 years	31	3.8	21	3.1	10	7.2	32.3	
35–44 years	108	13.4	87	13.0	21	15.1	19.4	
45–54 years	164	20.3	132	19.7	32	23.0	19.5	
55–64 years	190	23.5	156	23.3	34	24.5	17.9	
65–74 years	180	22.3	154	23.0	26	18.7	14.4	
75 years or older	126	15.6	114	17.0	12	18.6	9.5	
Total	808	100.0	669	100.0	139	100.0	17.2	

Family household								
Alone	84	10.4	69	10.3	15	10.8	17.9	0.121
With wife/husband or partner	317	39.2	262	39.2	55	39.6	17.4	
With wife/husband or partner and sons/daughters	244	30.2	192	28.7	52	37.4	21.3	
With sons/daughters	62	7.7	54	8.1	8	5.8	12.9	
In elderly care homes	2	0.2	2	0.3	—	—	—	
Other	99	12.3	90	13.5	9	6.5	9.1	
Total	808	100.0	669	100.0	139	100.0	17.2	

Professional/occupational status								
Full or part-time worker	242	30.0	180	26.9	62	44.6	25.6	<**0.001**
Student	6	0.7	5	0.7	1	0.7	16.7	
Unemployed	101	12.5	84	12.6	17	12.2	16.8	
House worker or domestic worker	33	4.1	26	3.9	7	5.0	21.2	
Retired or preretired	387	47.9	342	51.1	45	32.0	11.6	
Other	39	4.8	32	4.8	7	5.0	17.9	
Total	808	100.0	669	100.0	139	100.0	17.2	

Education level								
No education	24	3.0	24	4.1	—	—	—	0.195
1–4 years (basic 1st cycle)	401	49.8	330	56.4	71	51.1	17.7	
5–9 years (basic 2nd and 3rd cycles)	189	23.4	156	26.7	33	23.7	17.5	
10–12 years (secondary)	85	10.5	66	11.3	19	13.7	22.4	
More than 12 years (higher)	86	10.7	74	12.6	12	8.6	14.0	
Other	21	2.6	17	2.6	4	2.9	19.0	
Total	806	100.0	667	100.0	139	100.0	17.2	

Each row of the table includes absolute (*n*) and relative (%) frequencies of each characteristic and the utilization frequency (%) of interventional pain management by category (UF). Highlighted in bold are statistically significant results, at a 0.05 significance level. CP, chronic pain. IPM, interventional pain management. P25–P75, 25th percentile and 75th percentile (representing the interquartile range).^*∗*^*p* value for statistical hypothesis tests comparing the subsample of CP subjects with IPM to CP subjects without IPM. Chi-square test or Fisher's exact test for categorical variables and Mann–Whitney test for numerical variables were used. ^†^Chronic pain was defined, using the IASP standard definition, as pain present with duration ≥3 months.

**Table 3:** Clinical characteristics of all patients, chronic pain patients with interventional pain management, and chronic pain patients without interventional pain management.

Clinical characteristics of pain	All ^†^CP patients		CP patients without IPM		CP patients with IPM			^*∗*^ *p*
	*n*	%	*n*	%	*n*	%	UF (%)	

Duration (categorized)								
≤3 months–≤1 year	218	27.3	196	29.7	22	15.8	10.1	**0.006**
<1-≤2 years	110	13.8	87	13.2	23	16.5	20.9	
<2–≤5 years	157	19.6	128	19.4	29	20.9	18.5	
<5–≤10 years	123	15.4	103	15.6	20	14.4	16.3	
>10 years	191	23.9	146	22.1	45	32.4	23.6	
Total	799	100.0	660	100.0	139	100.0	17.4	

^‡^Persistence pattern								
Continuous	754	93.4	626	93.7	128	92.1	17.0	0.455
Discontinuous	53	6.6	42	6.3	11	7.9	20.8	
Total	807	100.0	668	100.0	139	100.0	17.2	

	Mean (SD)		Mean (SD)		Mean (SD)			

Intensity								
Pain on average (0–10 NRS)	5.96	(2.13)	6.00	(2.01)	5.77	(2.50)		0.641
Pain at its least (0–10 NRS)	3.63	(2.50)	3.63	(2.44)	3.63	(2.79)		0.759
Pain at its worst (0–10 NRS)	7.88	(2.11)	8.01	(1.98)	7.23	(2.61)		**0.003**
Pain right now (0–10 NRS)	4.83	(2.99)	4.88	(2.94)	4.58	(3.23)		0.304

	*n*	%	*n*	%	*n*	%	UF (%)	

No pain	14	1.8	6	0.9	8	6.2	57.1	<**0.001**
^§^Mild	229	29.4	181	27.8	48	37.2	21.0	
^§^Moderate	244	31.3	215	33.0	29	22.5	11.9	
^§^Severe	293	37.6	249	38.2	44	34.1	15.0	
Total	780	100.0	651	100.0	129	100.0	16.5	

^||^Location								
Lower limb	742	34.4	590	33.7	152	38.7	20.5	
Dorsum and lower back	414	19.3	343	19.6	71	18.1	17.1	
Upper limb	363	16.9	288	16.4	75	19.1	20.7	
Cervical and facial region	261	12.1	224	12.8	37	9.4	14.2	**0.047**
Bones/joints/muscles- unspecified	89	4.1	82	4.7	7	2.3	7.9	
Abdominal and anterior thoracic region	87	4.0	78	4.4	9	6.1	10.3	
Inguinal and pelvic region	77	3.6	53	3.0	24	4.6	31.2	
Other	115	5.6	97	5.5	18	4.6	15.7	
Total	2150	100.0	1757	100.2	393	100.0	18.3	

	Mean (SD)		Mean (SD)		Mean (SD)			

Pain-related interference								
General activity	6.90	(2.82)	7.04	(2.71)	6.20	(3.26)		**0.015**
Mood	6.25	(3.28)	6.37	(3.21)	5.64	(3.58)		0.054
Walking ability	6.52	(3.51)	6.67	(3.44)	5.76	(3.81)		**0.024**
^¶^Normal work	7.06	(2.98)	7.26	(2.82)	6.09	(3.53)		**<0.001**
Relations with other people	4.05	(3.60)	4.22	(3.58)	3.17	(3.64)		**0.002**
Sleep	5.64	(3.80)	5.72	(3.81)	5.20	(3.74)		0.087
Enjoyment of life	5.33	(3.84)	5.55	(3.79)	4.18	(3.95)		**<0.001**

	*n*	%	*n*	%	*n*	%	UF (%)	

^£^Interference								
No interference	14	1.8	6	0.9	8	6.3	57.1	<**0.001**
Mild	229	29.4	180	27.6	49	38.3	21.4	
Moderate	244	31.3	208	31.9	36	28.1	14.8	
Severe	293	37.6	258	39.6	35	27.3	11.9	
Total	780	100.0	652	100.0	128	100.0	16.4	

General health								
Excellent	13	1.6	11	1.7	2	1.4	15.4	
Very good	15	1.9	12	1.8	3	2.2	20.0	
Good	117	14.6	94	14.1	23	16.5	19.7	0.082
Fair	342	42.5	271	40.8	71	51.1	20.8	
Poor	317	39.4	277	41.7	40	28.8	12.6	
Total	804	100.0	665	100.0	139	100.0	17.3	

Each row of the table includes the absolute (*n*) and relative (%) frequencies of each characteristic, the utilization frequency (%) of interventional pain management by category (UF (%)), and in some cases the mean and standard deviation (SD). Highlighted in bold are statistically significant results, at a 0.05 significance level. CP, chronic pain. IPM, interventional pain management. SD, standard deviation. ^*∗*^*p* value for statistical hypothesis tests comparing the subsample of CP subjects with IPM to CP subjects without IPM. Chi-square test or Fisher's exact test for categorical variables and Mann–Whitney test for numerical variables were used. ^†^Chronic pain was defined, using the IASP standard definition, as pain present with duration ≥3 months. ^‡^Persistence pattern: continuous—pain present every day/ always; discontinuous—recurrent (pain present once to several times/ week); and sporadic (pain present less than once to several times/month). ^§^Pain intensity score was categorized taking into account the Serling classification: mild (1–4), moderate (5-6), and severe pain intensity (7–10). ^||^Multiple pain locations were recorded for each patient. ^¶^Including both work outside the home and housework. ^£^Pain interference was categorized as mild (1–4), moderate (5-6), and severe pain interference (7–10).

**Table 4:** Prescriptions and rate of prescription of interventional pain management and other therapeutic procedures performed on-site at multidisciplinary chronic pain clinics at baseline, six months, and twelve months and during the whole follow-up period.

	Baseline (*nt* = 808)		Six months (*nt* = 801)		Twelve months (*nt* = 744)		Total throughout the one-year follow-up (*nt* = 744)	
	*n*	Rate	*n*	Rate	*n*	Rate	*n*	Rate
Interventional pain management								
Nerve blocks	41	5.1	51	6.4	37	5.0	108	14.5
Viscosupplementation	19	2.4	6	0.7	4	0.5	22	3.0
Radiofrequency	12	1.5	16	2.0	5	0.7	31	4.2
Botulinum toxin	10	1.2	2	0.2	3	0.4	14	1.9
Epidural injections	9	1.1	11	1.4	2	0.3	19	2.6
Neurostimulation	—	—	1	0.1	—	—	1	0.1
Total	91	11.3	87	10.9	51	6.9	195	26.2
OTP performed on-site								
Infusions	43	5.3	38	4.7	25	3.4	95	12.8
Acupuncture	38	4.7	73	9.1	45	6.0	128	17.2
Mesotherapy	1	0.1	1	0.1	—	—	2	0.3
Total	82	10.1	199	14.0	70	9.4	225	30.2

Each row of the table includes the number of interventional pain management or other therapeutic procedures performed on-site at multidisciplinary chronic pain clinics at each assessment time point and during the follow-up time (*n*), the average number of prescriptions per 100 patients at each assessment time point (rate). *nt*, number of patients at each assessment time point. OTP, other therapeutic procedures.

**Table 5:** Characterization of interventional pain management and other therapeutic procedures performed on-site at multidisciplinary chronic pain clinics by the five main diagnoses.

*Interventional pain management*	*ICD-10 diagnostic classification*				
Nerve blocks	Low back pain	Chronic postoperative pain	Low back pain with sciatica	Shoulder pain	Cervicalgia

np (%)	23 (25.7)	14 (15.4)	9 (9.9)	5 (5.5)	5 (5.5)
ni (%)	32 (20.9)	22 (14.4)	12 (7.8)	7 (4.6)	5 (3.3)

Viscosupplementation	Knee pain	Unspecified osteoarthritis, unspecified site	Chronic postoperative pain	Pain in unspecified joint	Low back pain with sciatica

np (%)	13 (68.4)	3 (15.8)	1 (5.3)	1 (5.3)	1 (5.3)
ni (%)	22 (75.9)	3 (10.3)	1 (3.4)	2 (6.9)	1 (3.4)

Botulinum toxin	Pelvic and perineal pain	Central pain syndrome	Chronic postprocedural pain	Cancer pain	Lower abdominal pain, unspecified

np (%)	9 (69.2)	1 (7.7)	1 (7.7)	1 (7.7)	1 (7.7)
ni (%)	11 (73.3)	1 (6.7)	1 (6.7)	1 (6.7)	1 (6.7)

Radiofrequency	Low back pain	Low back pain with sciatica	Shoulder pain	Knee pain	Other hereditary and idiopathic neuropathies

np (%)	7 (25.0)	5 (17.9)	3 (10.7)	3 (10.7)	2 (7.1)
ni (%)	9 (27.3)	5 (15.2)	3 (9.1)	3 (9.1)	2 (6.1)

Epidural injections	Low back pain	Low back pain with sciatica	Hip pain	Chronic postoperative pain	Radiculopathy, lumbar region

np (%)	5 (26.3)	4 (21.1)	2 (10.5)	2 (10.5)	2 (10.5)
ni (%)	5 (22.7)	4 (18.2)	2 (9.1)	2 (9.1)	3 (16.6)

Neurostimulation	Chronic postoperative pain				

np (%)	1 (100.0)				
ni (%)	1 (100.0)				

*OTP performed on-site*	*ICD-10 diagnostic classification*				

Infusions	Cancer pain	Disorders of trigeminal nerve	Chronic postoperative pain	Radiculopathy, lumbar region	Cervicalgia

np (%)	22 (57.9)	4 (10.5)	3 (7.9)	2 (5.3)	2 (5.3)
ni (%)	37 (45.1)	26 (31.7)	5 (6.1)	13 (15.9)	13 (15.9)

Acupuncture	Low back pain	Cervicalgia	Myalgia	Pain in unspecified joint	Fibromyalgia

np (%)	15 (23.1)	11 (16.9)	6 (9.2)	5 (7.7)	5 (7.7)
ni (%)	49 (30.4)	12 (7.5)	27 (16.8)	5 (3.1)	22 (13.7)

Mesotherapy	Low back pain	Unspecified mononeuropathy of lower limb			

np (%)	1 (50.0)	1 (50.0)			
ni (%)	1 (50.0)	1 (50.0)			

Each row of the table includes the number of patients and percentage (%) that were prescribed interventional treatments or other therapeutic procedures performed on-site at multidisciplinary chronic pain clinics (np) and the number and percentage (%) of prescribed interventional treatments or other therapeutic procedures performed on-site at multidisciplinary chronic pain clinics (ni). ICD, International Statistical Classification of Diseases and Related Health Problems. OTP, other therapeutic procedures.
