# Supplementary material for: Interventional Pain Management in Multidisciplinary Chronic Pain Clinics: A Prospective Multicenter Cohort Study with One-Year Follow-Up
Source: Pain Res Treat. 2017 Oct 15;2017:8402413. doi: 10.1155/2017/8402413 (PMC5661079; doi:10.1155/2017/8402413)
Supplement: Supplementary file 1 — Supplement 1: Methodological flowchart of patients' selection. Supplement 2: Diagnostic classification of all patients, CP patients with IPM and CP patients without IPM. Supplement 3: Prescribed IPM and OTC performed on-site at multidisciplinary chronic pain clinics by multidisciplinary chronic pain clinics, at baseline, six months and twelve months. [file 8402413.f1.pdf]

Supplement 1 - Methodological flowchart of patients' selection for this study.

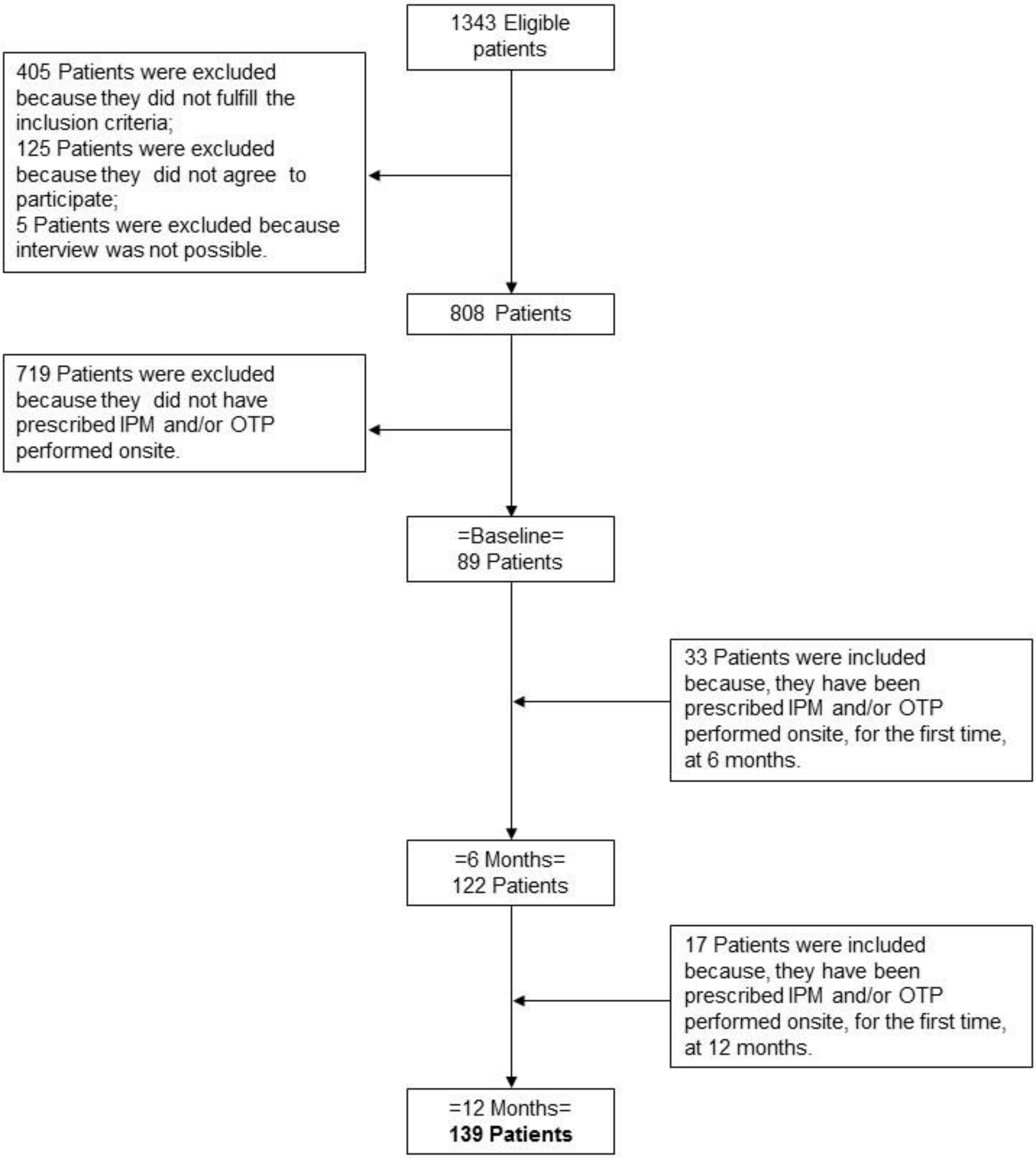

IPM, interventional pain management. OTP, other therapeutic procedures.

Supplement 2 - Diagnostic classification of all patients, chronic pain patients with interventional pain management and chronic pain patients without interventional pain management.

	All †CP patients		CP patients without IPM		CP patients with IPM			
	n	%	n	%	n	%	UF (%)	*p
**ICD-10 diagnostic classification**								
M54.5 - Low back pain	152	18.9	124	18.6	28	20.1	18.4	0.635
G89.3 - Cancer pain	118	14.6	113	16.9	5	3.6	4.2	**<0.001**
G89.28 - Chronic postoperative pain	70	8.7	54	8.1	16	11.5	22.9	0.187
M54.2 - Cervicalgia	53	6.6	48	7.2	5	3.6	9.4	0.135
M25.50 - Pain in unspecified joint	51	6.3	46	6.9	5	3.6	9.8	0.181
M54.16 - Radiculopathy, lumbar region	43	5.3	36	5.4	7	5.0	16.3	1.000
M25.56 - Knee pain	39	4.8	23	3.4	16	11.5	41.0	**<0.001**
M79.7 - Fibromyalgia	36	4.5	35	5.2	1	0.7	2.8	**0.013**
M54.4 - Low back with sciatica	30	3.7	19	2.8	11	7.9	36.7	**0.011**
R10.2 - Pelvic and perineal pain	28	3.5	15	2.2	13	9.4	46.4	**<0.001**
M19.90 - Unspecified osteoarthritis	27	3.3	20	3.0	7	5.0	25.9	0.295
M25.55 - Hip pain	19	2.4	13	1.9	6	4.3	31.6	0.117
I73 - Peripheral vascular diseases	18	2.2	18	2.7	-	-	-	-
M25.51 – Shoulder pain	18	2.2	12	1.8	6	4.3	33.3	0.104
B02.23 - Postherpetic polyneuropathy	17	2.1	17	2.5	-	-	-	-
G62.9 - Polyneuropathy, unspecified	15	1.9	15	2.2	-	-	-	-
M79.1 - Myalgia	12	1.5	9	1.3	3	3.2	25.0	0.443
G57.9 - Unspecified mononeuropathy of lower limb	11	1.4	9	1.3	2	1.4	18.2	1.000
G50 - Disorders of trigeminal nerve	7	0.9	7	1.0	-	-	-	-
R51 - Cephalgia	7	0.9	6	0.9	1	0.7	14.3	1.000
M79.6 - Pain in limb, hand, foot, fingers and toes	5	0.6	4	0.6	1	0.7	20.0	1.000
G56 - Mononeuropathies of upper limb	4	0.5	2	0.3	2	1.4	50.0	0.139
G60.8 - Other hereditary and idiopathic neuropathies	4	0.5	2	0.3	2	1.4	50.0	0.139
G89.0 - Central pain syndrome	4	0.5	3	0.4	1	0.7	25.0	0.531
G50.1 - Atypical facial pain	3	0.4	3	0.4	-	-	-	-
R07.2 - Precordial pain	3	0.4	3	0.4	-	-	-	-
G57 - Mononeuropathies of lower limb	2	0.2	2	0.3	-	-	-	-
M 54.3 - Sciatica	2	0.2	2	0.3	-	-	-	-
M79.63 - Forearm pain	2	0.2	2	0.3	-	-	-	-
R10.30 - Lower abdominal pain, unspecified	2	0.2	1	0.1	1	0.7	50.0	0.315
G52.1 - Disorders of glossopharyngeal nerve	1	0.1	1	0.1	-	-	-	-
G82.50 - Quadriplegia, unspecified	1	0.1	1	0.1	-	-	-	-
G90.50 - Complex regional pain syndrome I, unspecified	1	0.1	1	0.1	-	-	-	-
K86.1 - Other chronic pancreatitis	1	0.1	1	0.1	-	-	-	-

	‡806	100.0	667	100.0	139	100.0	17.2

In each row of the table: the absolute (n) and relative (%) frequencies of diagnostic classification and the utilization frequency (%) of interventional pain management by category (UF). Highlighted in bold are statistically significant results, at a 0.05 significance level. CP, chronic pain; ICD, International Statistical Classification of Diseases and Related Health Problems. *p- value for statistical hypothesis tests comparing the subsample of CP subjects with interventional management to CP subjects without interventional management. Fisher's exact test was used. †Chronic pain was defined, using the IASP standard definition, as pain present with duration ≥3 months. ‡2 incomplete medical records were found.

Supplement 3 - Prescribed interventional pain management and other therapeutic procedures performed onsite at multidisciplinary chronic pain clinics by multidisciplinary chronic pain clinics, at baseline, six months and twelve months.

**Interventional pain management**	Baseline		Six months		Twelve months		Total throughout the one year follow up	
	n	Rate	n	Rate	n	Rate	n	UF
**MCPC_A**	(np=201)		(np=190)		(np=183)			
Nerve blocks	2	1.0	6	3.2	4	2.2	12	9.5
Epidural injections	1	0.5	-	-	1	0.5	1	5.3
Total	3	1.5	6	3.2	5	2.7	13	3.0
**MCPC_B**	(np=201)		(np=198)		(np=196)			
Nerve blocks	36	17.9	38	9.0	27	13.8	80	63.5
Viscosupplementation	19	9.5	6	4.8	4	2.0	22	100.0
Radiofrequency	12	6.0	16	3.0	5	2.6	31	100.0
Epidural injections	7	3.5	10	1.8	-	-	16	84.2
Total	74	36.8	70	18.6	36	18.4	149	34.0
**MCPC_C**	(np=181)		(np=169)		(np=167)			
Botulinum toxin	10	5.5	2	1.2	3	1.8	14	100.0
Nerve blocks	3	1.7	7	4.1	6	3.6	16	12.7
Epidural injections	1	0.6	1	0.6	1	0.6	2	10.5
Neuroestimulation	-	-	1	0.6	-	-	1	100.0
Total	14	7.7	11	6.5	10	6.0	33	7.5

**OTP performed onsite**	n	Rate	n	Rate	n	Rate	n	UF
**MCPC_A**	(np=201)		(np=190)		(np=183)			
Acupuncture	2	1.0	6	3.2	13	7.1	10	7.8
Infusion	-	-	-	-	1	0.5	1	1.1
Total	2	1.0	6	3.2	14	7.7	11	2.5
**MCPC_B**	(np=201)		(np=198)		(np=196)			
Acupuncture	9	4.5	38	19.2	12	6.1	47	36.7
Infusion	-	-	1	0.5	2	1.0	1	1.1
Mesotherapy	1	0.5	1	0.5	-	-	2	100.0
Total	10	5.0	40	20.2	14	7.1	50	11.4
**MCPC_C**	(np=181)		(np=169)		(np=167)			
Infusion	43	23.8	37	21.9	19	11.4	88	94.6
Acupuncture	23	12.7	4	2.4	4	2.4	26	20.3
Total	66	36.5	41	24.3	23	13.8	114	26.0
**MCPC_D**	(np=225)		(np=206)		(np=199)			
Acupuncture	4	1.8	25	12.1	16	8.0	45	35.2
Infusion	-	-	-	-	3	1.5	3	3.2
Total	4	1.8	25	12.1	19	9.5	48	11.0

In each row of the table: the number of prescribed treatments by multidisciplinary chronic pain clinics named from A to D at each assessment time point or throughout the one year follow up (n), the average number of prescriptions per 100 patients at each assessment time point (Rate) and the utilization frequency (%) of interventional pain management other therapeutic procedures performed onsite at multidisciplinary chronic pain clinics by category throughout the one year follow up (UF). MCPC, multidisciplinary chronic pain clinics. np, number of patients at each assessment time point. OTC, other therapeutic procedures.
